# Occult Hepatic Pathology in Medicolegal Autopsies: A Single-Center Histopathological Study From Jharkhand, India

**DOI:** 10.7759/cureus.113846

**Published:** 2026-08-03

**Authors:** Sujit D. R Biswal, Manoj K Paswan, Anshu Jamaiyar

**Affiliations:** 1 Pathology, Rajendra Institute of Medical Sciences, Ranchi, IND

**Keywords:** autopsy pathology, chronic hepatitis, forensic pathology, hepatic necrosis, hepatic steatosis, histopathological examination, liver diseases, liver histopathology, medicolegal autopsy, tertiary care centre

## Abstract

Background

The liver is a vital, highly vascular organ responsible for metabolism and is frequently affected by toxins, infections, circulatory disturbances, and systemic diseases. Medicolegal autopsies allow identification of both observable and clinically silent hepatic pathology, thereby aiding determination of the cause of death and characterization of local disease patterns.

Objective

To evaluate the histopathological spectrum of hepatic lesions in liver tissue obtained at medicolegal autopsy and to describe the frequency of clinically occult liver pathology within this autopsy series.

Methods

This was a retrospective observational study that included 196 liver tissue specimens received during medicolegal autopsies over a period of 18 months at a single tertiary care center in Jharkhand, India. All tissues were fixed in 10% neutral buffered formalin, processed, and stained with hematoxylin and eosin, and examined by light microscopy. Histopathological findings were recorded and analyzed using Jamovi 2.17.7 (The Jamovi Project, Sydney, Australia). The chi-square or Fisher's exact test was used for categorical data and Student's independent-samples t-test for continuous data, with p < 0.05 considered significant.

Results

Of 196 cases, 167 (85.2%) were male and 29 (14.8%) female. Mean liver weight was higher in males (1413.28 g) than in females (902.90 g). The most common gross findings were fatty change (92/196, 46.9%) followed by congestion (55/196, 28.1%). On histopathology, steatosis (98/196, 50.0%) and morphological chronic hepatitis (88/196, 44.9%) were the most frequent findings, followed by congestion (32/196, 16.3%) and necrosis (21/196, 10.7%). Granulomas and tumors were rare.

Conclusion

Medicolegal autopsy-based histopathological examination revealed a substantial burden of clinically silent hepatic lesions, particularly steatosis and chronic hepatitis, within this selected medicolegal cohort. These findings underscore the continuing value of autopsy histopathology, even in the era of advanced imaging and molecular diagnostics, for detecting occult liver disease. Given the selected, single-center and trauma-predominant nature of the cohort, the observed frequencies should be interpreted as hypothesis-generating rather than as population prevalence estimates.

## Introduction

The liver is a metabolically active and highly vascular organ that is affected by a wide spectrum of pathological insults, including infections, toxins, circulatory disturbances, metabolic disorders, and systemic diseases [1,2]. Because of its central role in metabolism and detoxification, liver injury may remain clinically silent for prolonged periods and becomes evident only during histopathological examination [2,3]. Recently, the global burden of chronic liver diseases, especially metabolic dysfunction-associated steatotic liver disease (MASLD) and chronic hepatitis, has increased significantly, posing a major public health concern [2]. Autopsy continues to play an important role in identifying clinically unsuspected diseases, determining the cause of death, and evaluating underlying pathological processes [1-4]. Although in this modern era, we have observed recent advancements in the field of diagnostic imaging, laboratory medicine, and molecular diagnostics, discrepancies between antemortem clinical diagnoses and autopsy findings continue to exist [5]. Hence, histopathological examination of autopsies remains an important tool not only for medical audit but also for understanding disease patterns within a population [3,5]. Medicolegal autopsies help in evaluating occult liver pathology in an unselected population, particularly in cases of sudden, traumatic, or unexplained deaths [6-8]. Such examinations help in revealing clinically undiagnosed liver diseases, which usually remain unidentified during life. Autopsy-based studies help in providing insights into the background burden of chronic liver diseases and associated metabolic disorders for developing regions having limited healthcare access and inadequate population-based epidemiological data [7,8]. Histopathological analyses of liver tissues obtained from medicolegal autopsies contribute to determining pathological changes associated with death and identifying underlying silent liver diseases, including steatosis, chronic hepatitis, fibrosis, necrosis, granulomatous lesions, and tumors [3,9]. Understanding the pattern of liver lesions in medicolegal autopsies may provide region-specific insights and help in understanding the burden of occult liver disease, while recognizing that a medicolegal series is a selected population. Hence, the present study was conducted to describe the histopathological spectrum of liver lesions in medicolegal autopsies at a single tertiary care center in Jharkhand, eastern India, and to assess the frequency of clinically unsuspected hepatic disease in this autopsy population.

## Materials and methods

Study design and setting

This retrospective observational study was conducted in the Department of Pathology at Rajendra Institute of Medical Sciences (RIMS), Ranchi, Jharkhand, India, a single tertiary care teaching hospital and referral center in eastern India. The study was carried out over a period of 18 months, from February 2024 to July 2025. The objective was to describe the histopathological spectrum of hepatic lesions in medicolegal autopsy cases and to assess the frequency of clinically occult liver pathology in the study population.

Study population and eligibility criteria

A total of 196 liver tissue specimens obtained from medicolegal autopsy cases were included in this study. Adequately preserved specimens suitable for histopathological examination, with complete demographic and medicolegal records, were included. Autolyzed specimens interfering with histopathological examination, cases with inadequate tissue sampling, improper fixation, and incomplete records were excluded. Because the cohort comprised only medicolegal autopsies, it represents a selected (convenience) population rather than a random population sample.

Ethical approval and consent

This retrospective study was approved by the Institutional Ethics Committee of Rajendra Institute of Medical Sciences (RIMS), Ranchi, Jharkhand, India (IEC Registration No. ECR/769/INST/JH/2015/RR-21; Letter No. 87, dated 01/02/2025). All medicolegal autopsies were performed under statutory authority in accordance with institutional and governmental medicolegal protocols, and consent for the autopsy procedure had been obtained from the immediate relatives or legal guardians of the deceased at the time of autopsy, as per institutional policy. As the present study involved only the retrospective analysis of archived tissue blocks and anonymized medicolegal records, the Institutional Ethics Committee waived the requirement for additional individual informed consent. Confidentiality and anonymity of patient and medicolegal data were maintained throughout the study.

Autopsy procedure and gross examination

Autopsies were performed by the Department of Forensic Medicine, where the liver was examined in situ for size, capsular integrity, surface nodularity, discolouration, traumatic laceration, congestion, and gross fatty change. After removal, liver weight was measured using a calibrated digital weighing scale. Gross findings were documented systematically, including fatty change, congestion, traumatic laceration, nodularity, and green-yellow discolouration of the cut surface, and representative tissue sections were obtained from grossly abnormal areas.

Tissue fixation and histopathological processing

Liver tissue specimens were fixed in 10% neutral buffered formalin for approximately 24 hours at room temperature prior to processing. After fixation, tissue processing was carried out using standard histopathological techniques, including dehydration through graded alcohol concentrations, clearing with xylene, and paraffin wax infiltration. Tissues were subsequently embedded in paraffin blocks. Paraffin-embedded tissue blocks were sectioned at approximately 4-5 µm thickness using a rotary microtome, mounted on glass slides, and stained routinely with hematoxylin and eosin [2].

Histopathological evaluation

Microscopic evaluation of the liver tissues was performed under light microscopy at different magnifications. Histopathological parameters, including steatosis, chronic hepatitis, congestion, hepatocellular necrosis, granulomatous inflammation, fibrosis, cirrhotic changes, and neoplastic lesions, were evaluated. Steatosis was identified by the presence of intracellular fat vacuoles within hepatocytes causing cytoplasmic clearing and displacing nuclei. Chronic hepatitis was diagnosed morphologically based on portal inflammation, interface activity, and associated hepatocellular injury, without etiological subtyping. Congestion was identified by sinusoidal dilatation and vascular engorgement. Hepatocellular necrosis was identified by hepatocyte loss associated with eosinophilic cytoplasmic change and nuclear fragmentation or disappearance. Granulomatous inflammation was diagnosed by the presence of epithelioid granulomas with or without multinucleated giant cells. Cirrhotic changes were identified based on the nodular architectural distortion associated with fibrosis. Neoplastic lesions were diagnosed based on abnormal cellular proliferation and cytological atypia. Owing to the retrospective design, variability in tissue sampling, and absence of clinical correlation, lesions were recorded as present or absent; formal semiquantitative grading and staging systems (for example, the NAS (NAFLD Activity Score), SAF (Steatosis-Activity-Fibrosis), METAVIR (Meta-analysis of Histological Data in Viral Hepatitis), or Ishak systems) could not be uniformly applied and were therefore not used. More than one histopathological lesion was allowed to coexist in individual cases, and each lesion was recorded independently for statistical analysis.

Clinicopathological correlation

Histopathological findings were correlated with demographic profile, liver weight, gross examination, manner of death, and cause of death. Manner of death was categorized as accidental, suicidal, homicidal, or natural. Cause of death was obtained from autopsy records and classified into major categories, including head injury, hemorrhagic shock, septicemia or infectious causes, asphyxial deaths, coronary artery disease, pulmonary causes, and miscellaneous causes. Gross findings were correlated with histopathological findings to assess concordance between macroscopic and microscopic assessment.

Statistical analysis

Data were entered into Microsoft Excel (Microsoft Corporation, Redmond, Washington, USA) and analyzed using Jamovi 2.17.7 (The Jamovi Project, Sydney, Australia). Continuous variables were expressed as mean ± standard deviation (SD) or median, whereas categorical variables were expressed as frequencies and percentages. Comparisons between categorical variables were performed using the chi-square test or Fisher's exact test. Student's independent-samples t-test was used for a comparison of continuous variables between groups. The Cochran-Armitage trend test was applied to evaluate age-related trends in chronic hepatitis frequencies across age categories. Associations between liver-weight groups and steatosis were analyzed using the chi-square test.

Multivariable binary logistic regression was used to identify independent predictors of steatosis. Candidate covariates were restricted to variables reliably documented in the medicolegal records, namely, age, sex, and the presence of morphological chronic hepatitis, and were selected a priori based on biological plausibility and data availability rather than by automated stepwise selection. With 98 steatosis events and 3 covariates, the model satisfied the conventional threshold of at least 10 events per variable. Clinically relevant covariates, such as alcohol use, body mass index, diabetes status, viral hepatitis markers, and toxicology findings, were not available in the retrospective records and could not be included; the resulting estimates are therefore adjusted only for measured covariates and are interpreted as associations rather than causal effects. A two-sided p < 0.05 was considered statistically significant throughout.

## Results

**Figure 1:**
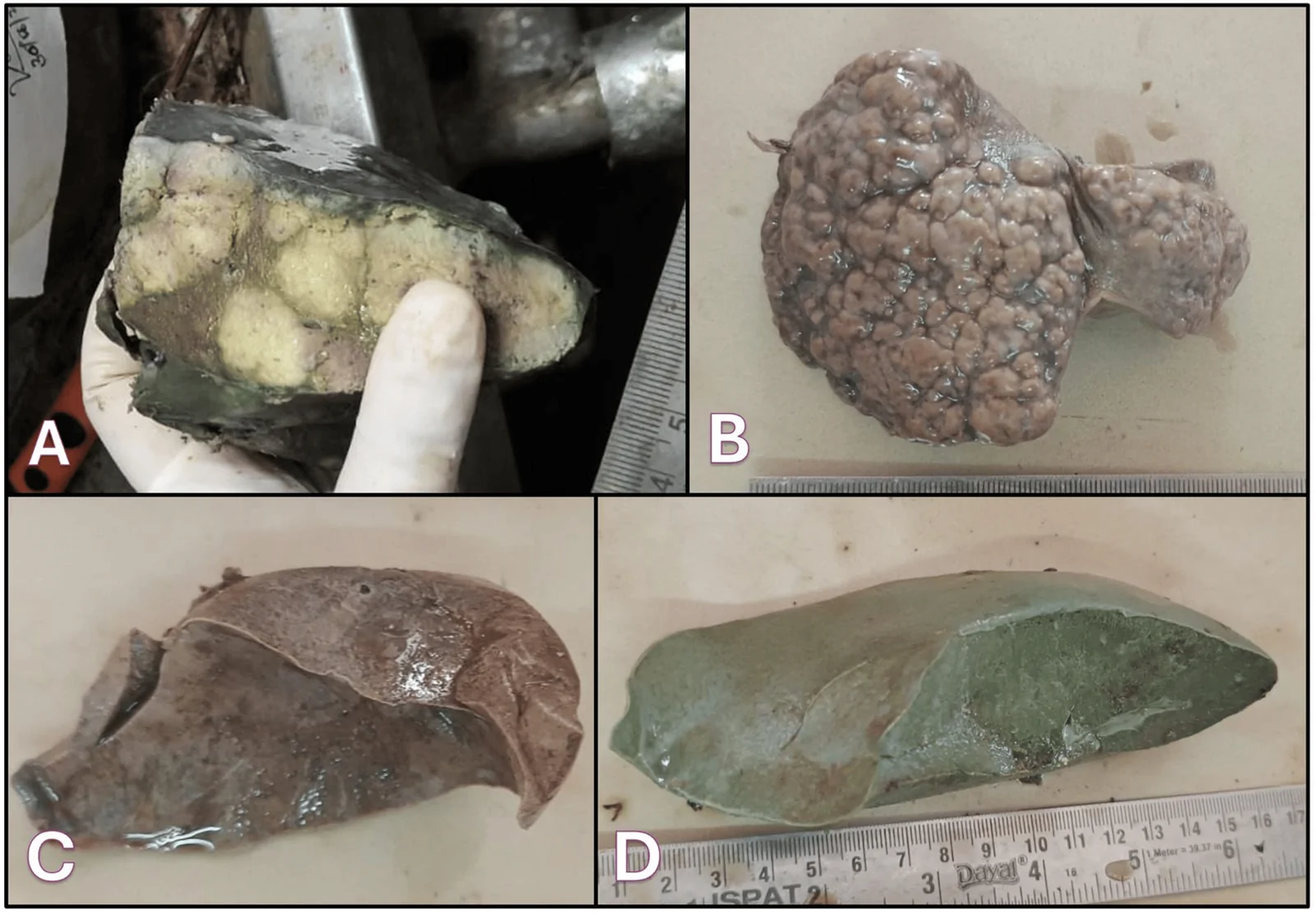
Gross hepatic pathologies in medicolegal autopsy specimens (A) Cut surface showing multiple, well-defined, yellowish-white space-occupying lesions replacing hepatic parenchyma. (B) External surface showing diffuse nodular "cobblestone" pattern consistent with macronodular cirrhosis. (C) Cut surface showing alternating congested and pale areas producing a "nutmeg liver" pattern, consistent with chronic venous congestion. (D) Cut surface showing diffuse greenish-yellow discoloration with greasy consistency, consistent with hepatic steatosis (ruler in cm for scale).

A total of 196 autopsy livers were examined, comprising 167 (85.2%) from male and 29 (14.8%) from female decedents (Figure 1). Hepatic morphometry differed appreciably by sex. The mean liver weight in males was 1413.28 g (median 1249.0 g), substantially exceeding that of females at 902.90 g (median 901.0 g) (Table 1). The male distribution was right-skewed and widely dispersed (SD 425.41 g; range 969-2654 g), whereas female weights were narrowly clustered (SD 32.36 g; range 847-965 g). The majority of livers (130/196, 66.3%) fell within the 967-1600 g reference interval, while 37 (18.9%) exceeded 1600 g and 29 (14.8%) weighed below 967 g.

**Table 1:** Liver weight distribution by sex

Sex	n (%)	Mean (g)	Median (g)	SD (g)	Range (g)
Male	167 (85.2%)	1413.28	1249	425.41	969-2654
Female	29 (14.8%)	902.9	901	32.36	847-965

**Figure 2:**
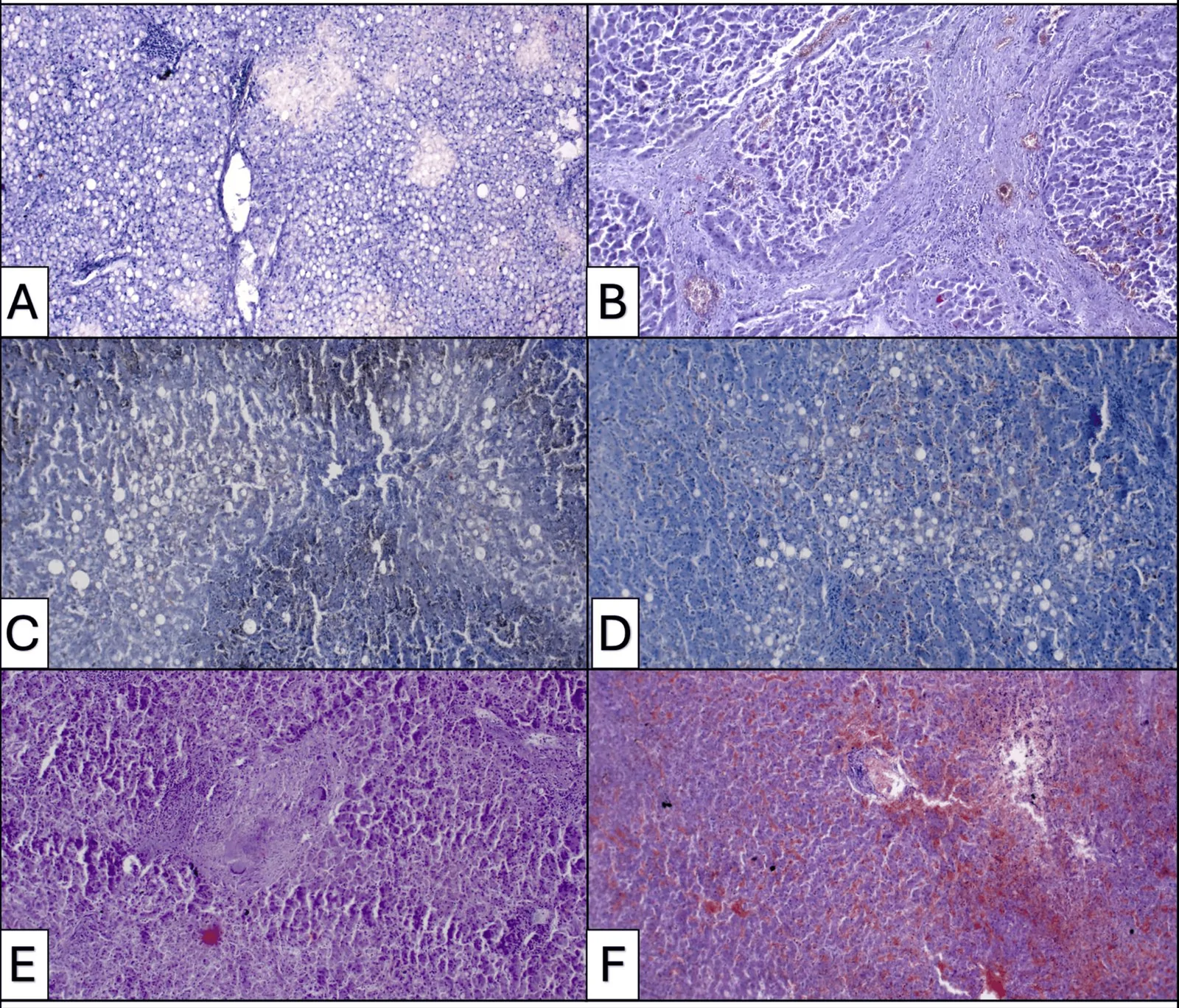
Microscopic (H&E, ×100) sections showing the spectrum of hepatic pathology in medicolegal autopsy specimens (A) Hepatocytes showing macrovesicular steatosis with large intracytoplasmic fat vacuoles displacing the nucleus, consistent with fatty liver (H&E, ×100). (B) Photomicrograph showing regenerative hepatocyte nodules separated by broad fibrous septa, consistent with cirrhosis (H&E, ×100). (C, D) Sections showing diffuse macrovesicular steatosis involving hepatocytes throughout the parenchyma, confirming fatty liver (H&E, ×100). (E) Photomicrograph showing a well-formed granulomatous lesion composed of epithelioid cells within the hepatic parenchyma (H&E, ×100). (F) Sinusoids diffusely dilated and engorged with red blood cells, consistent with hepatic congestion (H&E, ×100).

**Figure 3:**
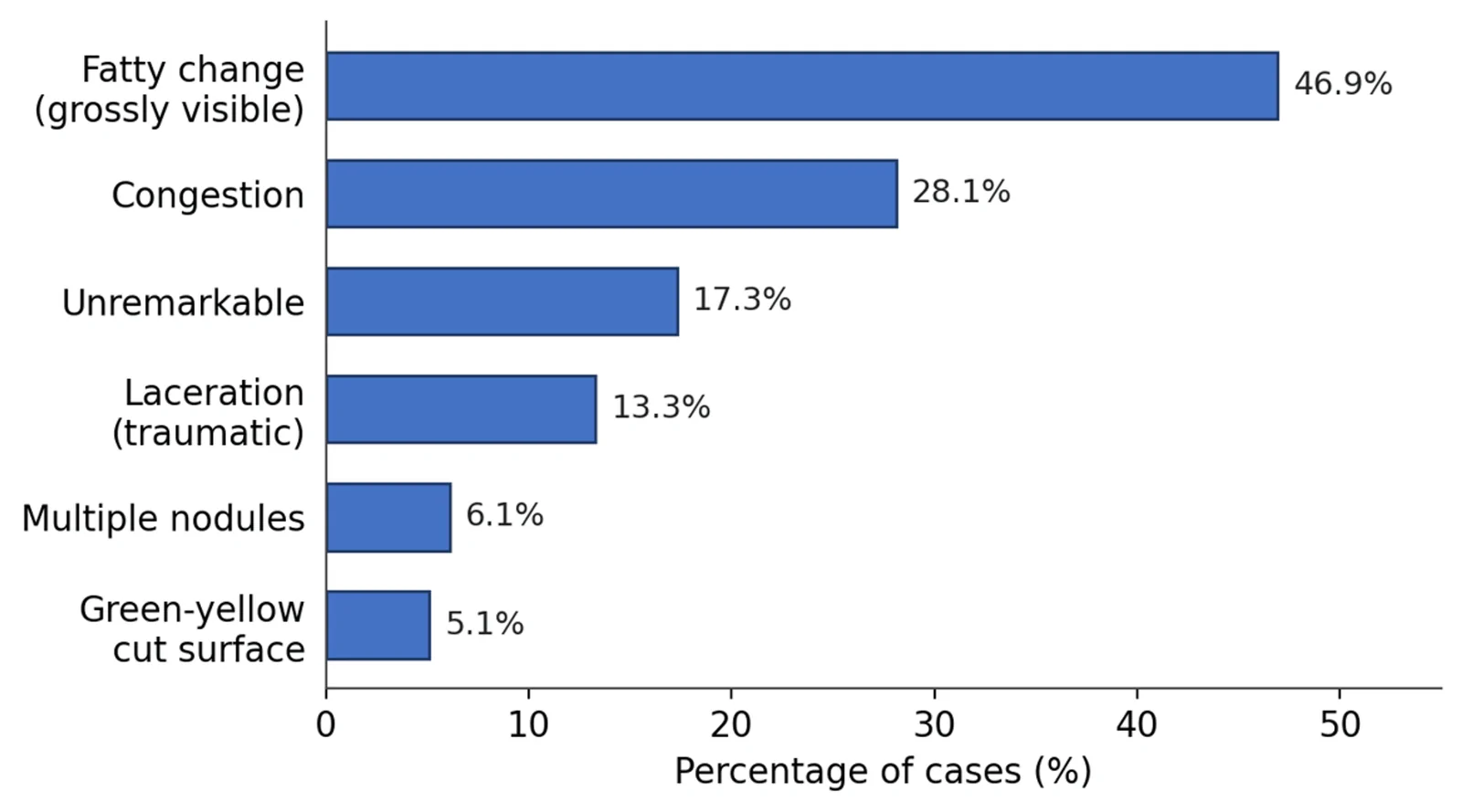
Spectrum of gross pathological findings (N = 196; findings non-mutually-exclusive)

On macroscopic examination, grossly visible fatty change was the most frequent finding (92/196, 46.9%), followed by congestion (55/196, 28.1%), with 34/196 (17.3%) of livers appearing unremarkable (Figure 2). Macroscopically suspicious lesions correlated poorly with neoplasia: among 4 space-occupying lesions and 12 nodular livers, only a single space-occupying lesion was confirmed as a tumor on histology, the remainder representing regenerative or cirrhotic nodularity (Figure 3). Concordance between gross and microscopic assessment was therefore finding-dependent, being high for steatosis (90% agreement; Cohen's κ = 0.622, substantial) but low for nodular-to-neoplastic correlation (8%).

Histologically, steatosis was the predominant lesion, present in exactly half the cohort (98/196, 50.0%), closely followed by morphological chronic hepatitis (88/196, 44.9%); congestion, necrosis, granuloma, and tumor were progressively less frequent (Table 2). Co-existing pathology was common: steatosis and chronic hepatitis occurred together in 52/196 (26.5%) of cases (the single most frequent combination), steatosis and congestion in 21/196 (10.7%), and hepatitis and necrosis in 15/196 (7.6%), while a triple-lesion phenotype was identified in 9 cases (4.6%).

**Table 2:** Frequency of histopathological findings (N = 196; findings non-exclusive)

Histopathological finding	n	%
Steatosis	98	50
Chronic hepatitis (mild/severe)	88	44.9
Chronic venous congestion/congestion	32	16.3
Necrosis	21	10.7
Granuloma	3	1.5
Tumor	1	0.5

**Figure 4:**
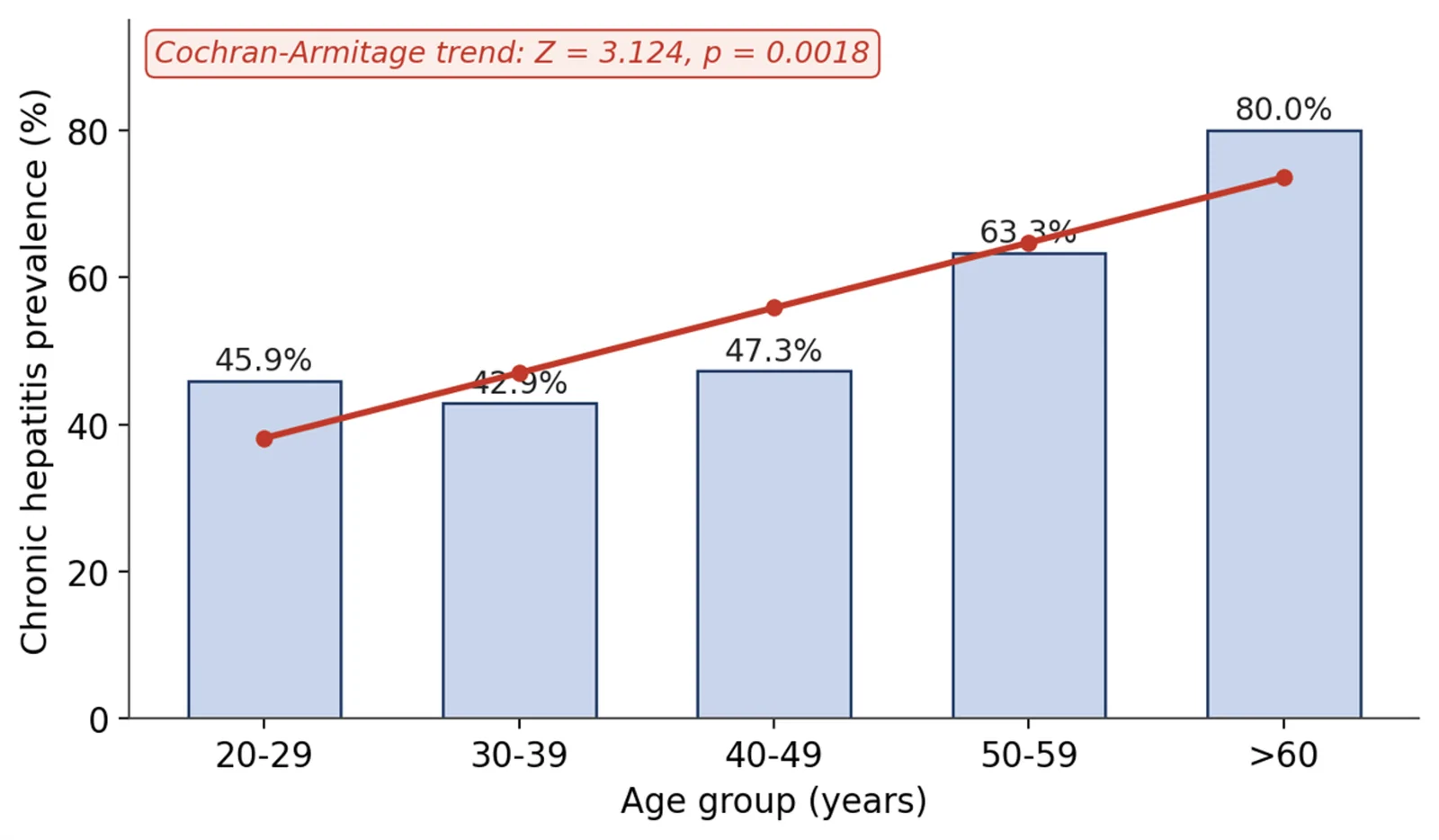
Chronic hepatitis prevalence across age groups, with the fitted linear trend (Cochran-Armitage trend test, p = 0.0018)

The frequency of the principal lesions did not differ significantly between the sexes. Steatosis, chronic hepatitis, and necrosis were comparably distributed in males and females on Fisher's exact testing (Table 3). Given the small female subgroup (n = 29), these sex-stratified comparisons are underpowered and should be regarded as exploratory and hypothesis-generating. Age, however, exerted a clear effect on chronic hepatitis, the frequency of which rose progressively from 45.9% in those aged 20-29 years to 80.0% in those older than 60 years, a statistically significant linear trend on the Cochran-Armitage test (Z = 3.124; p = 0.0018) (Figure 4).

**Table 3:** Histopathological findings by sex (Fisher's exact test)

Histopathological finding	n	%
Steatosis	98	50
Chronic hepatitis (mild/severe)	88	44.9
Chronic venous congestion/congestion	32	16.3
Necrosis	21	10.7
Granuloma	3	1.5
Tumor	1	0.5

**Figure 5:**
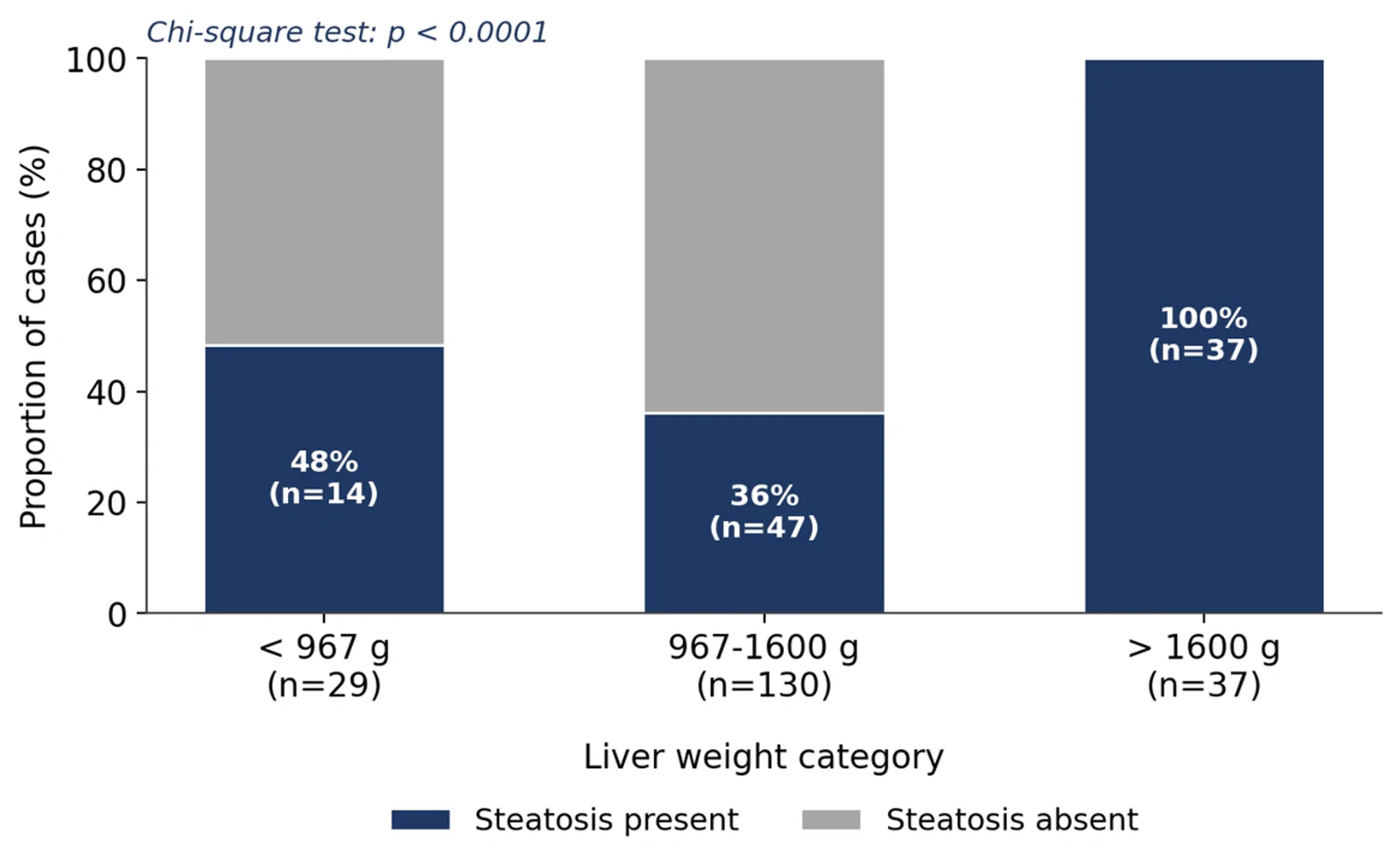
Steatosis status by liver-weight category, shown as the proportion of cases with and without steatosis (chi-square test, p < 0.0001)

Steatosis showed a strong, graded association with liver weight, its frequency rising to 100% in livers exceeding 1600 g (chi-square test, p < 0.0001) (Figure 5), and a positive correlation between absolute liver weight and steatosis was confirmed on both parametric and non-parametric testing (Pearson r = 0.36, p < 0.001; Spearman ρ = 0.218, p = 0.002). On multivariable logistic regression, increasing age and the presence of morphological chronic hepatitis were each independently and inversely associated with steatosis, whereas male sex was not a significant predictor (Table 4). As noted in the Methods, these estimates are adjusted only for the covariates available in the medicolegal records and should be interpreted as associations rather than causal effects.

**Table 4:** Multivariable logistic regression for predictors of steatosis

Variable	Odds Ratio (OR)	95% Confidence Interval (CI)	p-value
Male sex	0.88	0.37-2.04	0.758
Age	0.97	0.95-0.99	0.016*
Chronic hepatitis	0.33	0.18-0.60	<0.001*

Correlation with manner and cause of death

**Figure 6:**
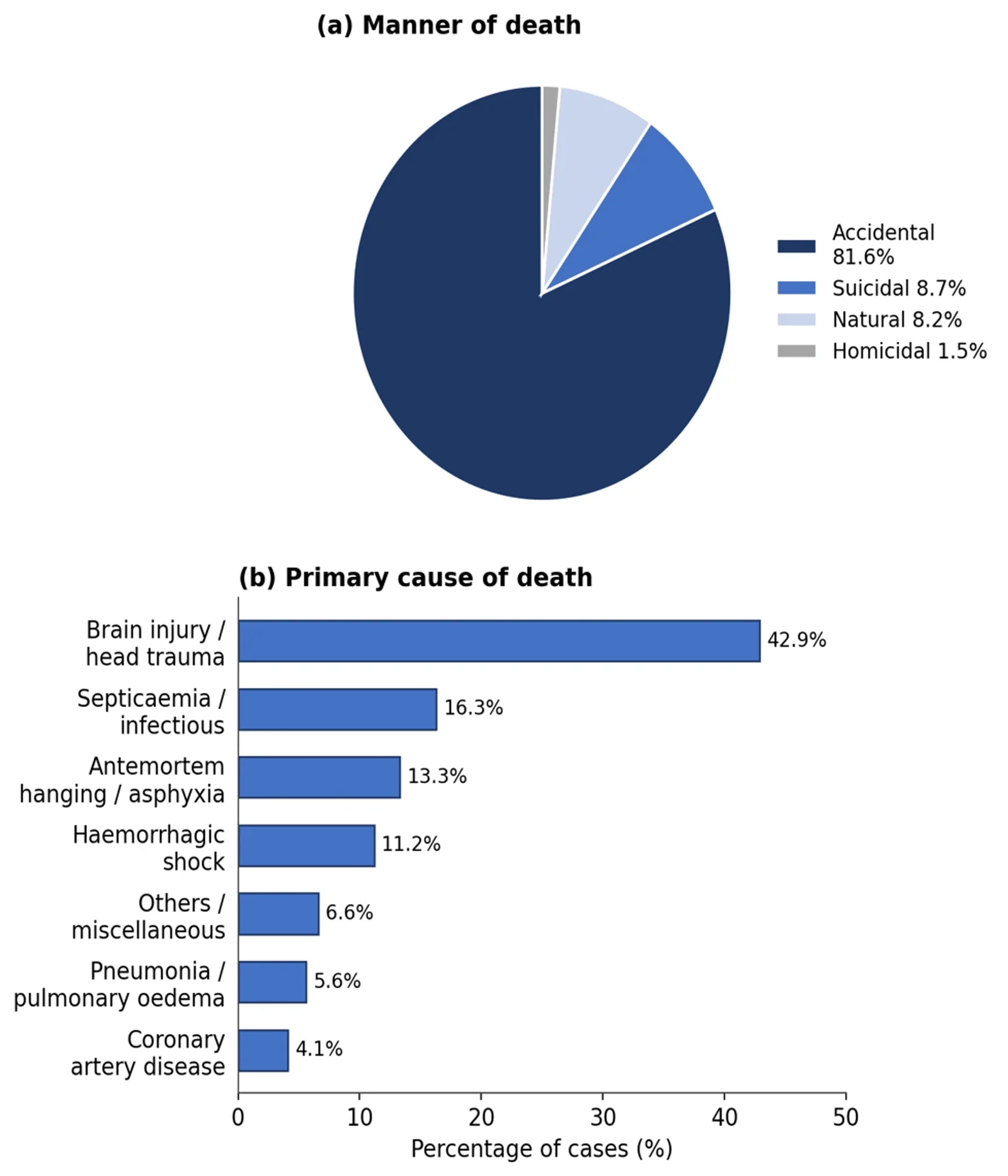
Mortality profile of the cohort: (a) manner of death and (b) primary cause of death

The series was dominated by deaths of accidental manner (160/196, 81.6%), with brain injury and head trauma the leading primary cause of death (84/196, 42.9%), ahead of septicemia (32/196, 16.3%) and asphyxial deaths (26/196, 13.3%) (Figure 6). To assess whether the hepatic lesions were specific to the mode of dying, their distribution was tested against the manner of death: neither steatosis nor chronic hepatitis was significantly associated with the manner of death (chi-square = 6.36, p = 0.095, and chi-square = 4.58, p = 0.205, respectively). This lack of association indicates that the hepatic pathology identified reflected background, population-level disease rather than lesions specific to any particular cause or manner of death.

## Discussion

The present study describes the histopathological spectrum of hepatic lesions in medicolegal autopsies from a single tertiary care center in Jharkhand, eastern India, and demonstrates a substantial burden of clinically occult hepatic pathology within this selected autopsy cohort. Histopathological examination revealed steatosis and chronic hepatitis as the predominant liver lesions, emphasizing the continued relevance of medicolegal autopsy in identifying previously undiagnosed liver disease and generating region-specific insights into the background burden of hepatic disease, while acknowledging that a medicolegal series is not representative of the general population [1-5]. The study population showed a marked male predominance, accounting for 85.2% of all cases. This predominance is an inherent characteristic of medicolegal autopsy cohorts rather than a sampling artefact, and similar observations have been reported in previous medicolegal autopsy studies, likely reflecting greater exposure of males to trauma, occupational hazards, alcohol consumption, and outdoor activities [10-12]. The majority of cases belonged to younger age groups, mostly the third and fourth decades of life, which reflects the predominance of accidental and trauma-related deaths in the medicolegal autopsy population. The higher mean liver weight among males in the present study is also comparable to previous autopsy-based organ weight studies in Indian populations [13,14].

Steatosis was the most common histopathological lesion in the present study, contributing 50.0% of cases. This finding is notable because the majority of cases were trauma-related autopsies rather than clinically diagnosed chronic liver disease. The high frequency of steatosis may reflect a considerable burden of occult metabolic liver disease within the underlying population. Similar observations have been recorded in studies evaluating non-alcoholic fatty liver disease (NAFLD) and MASLD, which are increasingly recognized as major global public health concerns [15-17]. A significant positive association was observed between liver weight and steatosis, with increasing liver weight associated with a greater frequency of steatosis; notably, all liver specimens weighing more than 1600 g showed histopathological evidence of steatosis. This finding supports the known association between hepatic fat accumulation and hepatomegaly and is consistent with the pathological basis of steatotic liver enlargement described in previous studies [15-17]. On multivariable logistic regression, chronic hepatitis showed a statistically significant inverse association with steatosis after adjustment for age and sex. Increasing age showed a modest inverse association with steatosis, while sex did not show a significant association. The inverse association between chronic hepatitis and steatosis may reflect different pathophysiological mechanisms underlying inflammatory and metabolic liver injury patterns within the study population. Such overlapping yet distinct injury pathways have been recognized in chronic liver disease and metabolic liver disorders [15-18]. Because clinical covariates, such as alcohol use, body mass index, diabetes, and viral serology, were unavailable, these associations may be affected by residual confounding and should be interpreted with caution.

The second most common finding was chronic hepatitis, contributing 44.9% of cases, which showed a statistically significant progressive increase in frequency with advancing age, reaching 80.0% among individuals older than 60 years. This age-related trend may reflect cumulative exposure to hepatotoxic insults, such as chronic alcohol consumption, metabolic dysfunction, environmental factors, chronic infection, and systemic diseases, over time [18-20]; however, the specific etiology could not be established in this study. A similar age-related increase in chronic hepatitis frequency has been documented previously [18-20]. Etiological classification of chronic hepatitis could not be performed because of the retrospective nature of the study and the lack of serological, toxicological, and clinical correlations. Nonetheless, the presence of portal inflammatory infiltrates, interface activity, and associated hepatocellular injury in a substantial proportion of cases indicates a considerable burden of morphological chronic inflammatory liver disease in the study population. The term chronic hepatitis in this study therefore denotes a morphological pattern rather than an etiologically defined clinical diagnosis.

Congestion and hepatocellular necrosis contributed 16.3% and 10.7% of cases, respectively. These findings are related to terminal circulatory disturbances, hypoxia, shock, septicemia, and fatal hemodynamic changes occurring prior to death [18,21]. Congestion is a frequent autopsy finding, occurring secondary to impaired venous return and terminal cardiovascular collapse. Similarly, hepatocellular necrosis occurs due to ischemic injury, toxic exposure, hypoxic hepatitis, or circulatory compromise [18,21]. Toxic liver injury due to poisoning, particularly aluminium phosphide toxicity, may additionally contribute to necrotic hepatic changes [22,23]. The least common findings in the present study were granulomatous inflammation and neoplastic lesions. Granulomatous lesions occur due to infectious etiologies such as tuberculosis and fungal infection, particularly in developing countries [24]. Only a single neoplastic lesion was identified histopathologically, even though gross nodularity was seen in multiple cases. Most gross nodules were either regenerative or cirrhotic changes rather than true neoplastic lesions. This finding highlights the limitations of gross examination alone and underscores the importance of microscopic evaluation for definitive characterization of hepatic lesions [3,9].

Gross examination demonstrated substantial agreement with the histopathological diagnosis of steatosis, with a Cohen's kappa coefficient of 0.622. This suggests that gross fatty change provides useful evidence of hepatic steatosis at autopsy; however, histopathological evaluation remains the gold standard for accurate and definitive diagnosis of hepatic lesions. In contrast, gross examination of nodularity demonstrated poor correspondence with neoplastic pathology, highlighting the limitations of macroscopic examination in differentiating regenerative, cirrhotic, and neoplastic hepatic nodules [3,9]. The majority of cases in the present study were accidental deaths associated predominantly with head injury and hemorrhagic shock. However, no statistically significant association was observed between the major histopathological findings and the manner of death, which suggests that the hepatic lesions identified microscopically largely represent underlying background hepatic pathology rather than direct cause-specific alterations. Similar findings have been reported in previous medicolegal autopsy studies evaluating incidental hepatic lesions [10]. The coexistence of multiple histopathological lesions within individual cases further emphasizes the multifactorial nature of liver injury; the most common combination was steatosis with chronic hepatitis. These overlapping patterns may reflect the interaction of metabolic dysfunction, chronic inflammation, alcohol-related injury, and circulatory disturbances occurring simultaneously within the liver parenchyma, and are increasingly recognized in chronic liver disease [15-20].

This study has several limitations that should be considered when interpreting its findings. First, it was a single-center, retrospective study conducted at one tertiary care institution, so its findings do not represent the true prevalence of hepatic lesions in the wider general population and are best regarded as within-cohort frequencies. Second, the predominance of trauma-related medicolegal autopsies introduced a selection bias toward younger male individuals, which limits generalizability across age and sex strata; in particular, with only 29 female decedents, all sex-stratified comparisons are underpowered and exploratory. Third, serological, toxicological, radiological, and molecular investigation data were unavailable, precluding etiological classification of chronic hepatitis and other liver lesions; special stains and immunohistochemistry were not performed owing to the retrospective design and limited availability of liver tissue. Consequently, the multivariable model could be adjusted only for the covariates recorded in the medicolegal files, and residual confounding by unmeasured factors, such as alcohol use, body mass index, diabetes, and viral status, cannot be excluded, so the reported associations should be read as associational rather than causal. Fourth, standardized grading and staging systems for steatosis, fibrosis, and chronic hepatitis could not be uniformly applied because of incomplete clinical correlation and variability in tissue sampling; lesions were therefore recorded as present or absent, which reduces comparability with graded series. In addition, two further methodological limitations are noted. Histopathological interpretation was performed by consultant pathologists, but inter-observer variation was not quantified using a kappa statistic, so a degree of subjective variability in lesion assessment cannot be excluded. Finally, although grossly autolyzed specimens were excluded, early post-mortem autolytic change may mimic or mask histological steatosis and mild inflammatory activity, potentially affecting the documented frequency of these findings. Despite these limitations, the present study provides useful zone-specific clinicopathological data on occult hepatic lesions in an underrepresented region of eastern India. Histopathological examination of liver tissue obtained at medicolegal autopsy remains a pivotal tool for identifying clinically silent liver disease, understanding regional disease patterns, and generating public health data that may support improved preventive strategies and healthcare planning [1-5,25].

## Conclusions

Histopathological examination of the liver in medicolegal autopsies reveals a spectrum of pathological changes, most of which are clinically unsuspected. In this cohort, steatosis and chronic hepatitis were the most common findings, reflecting the burden of metabolic and chronic liver disease within a selected medicolegal population. The presence of steatosis across all age groups points toward a growing metabolic health concern, consistent with metabolic dysfunction-associated steatotic liver disease (MASLD) and non-alcoholic fatty liver disease (NAFLD). The statistically significant age-related increase in chronic hepatitis may reflect the cumulative impact of hepatotoxic insults over the lifespan, although the etiology could not be determined in the present study. Congestion and necrosis were attributable to terminal physiological events, while traumatic lesions were related to the medicolegal causes of death. The integration of histopathological examination with medicolegal autopsy serves as an important tool not only for helping determine the cause of death but also for providing data-driven insights that may inform regional public health strategies and preventive interventions. Given the selected, single-center nature of the cohort, these findings should be regarded as hypothesis-generating and warrant confirmation in larger, multicenter studies.
